# Neuroprotective Effect of Arctigenin via Upregulation of P-CREB in Mouse Primary Neurons and Human SH-SY5Y Neuroblastoma Cells

**DOI:** 10.3390/ijms140918657

**Published:** 2013-09-10

**Authors:** Nan Zhang, Qingping Wen, Lu Ren, Wenbo Liang, Yang Xia, Xiaodan Zhang, Dan Zhao, Dong Sun, Yv Hu, Haiguang Hao, Yaping Yan, Guangxian Zhang, Jingxian Yang, Tingguo Kang

**Affiliations:** 1 School of Pharmacy, Liaoning University of Traditional Chinese Medicine, Dalian 116600, Liaoning, China; E-Mails: nanzhang2002@hotmail.com (N.Z.); zxd20060205009@aliyun.com (X.Z.); danzhao88@outlook.com (D.Z.); s43792211@sina.com (D.S.); cynthiafishu@aliyun.com (Y.H.); haiguang_hao@aliyun.com (H.H.); 2 First Affiliated Hospital, Dalian Medical University, Dalian 116011, Liaoning, China; E-Mail: wqp.89@163.com; 3 Liaoning University of Traditional Chinese Medicine, Shenyang 110847, Liaoning, China; E-Mail: renlu2008.student@sina.com; 4 Medical College of Dalian University, Dalian 116600, Liaoning, China; E-Mail: dllwb@126.com; 5 College of Engineering, University of California Berkeley, Berkeley, CA 94720, USA; E-Mail: xiayang9999@vip.sina.com; 6 Department of Neurology, Thomas Jefferson University, Philadelphia, PA 19107, USA; E-Mails: yaping.yan@snnu.edu.cn (Y.Y.); guang-xian.zhang@jefferson.edu (G.Z.)

**Keywords:** arctigenin, neuroprotection, beta amyloid (Aβ), p-CREB

## Abstract

Arctigenin (Arc) has been shown to act on scopolamine-induced memory deficit mice and to provide a neuroprotective effect on cultured cortical neurons from glutamate-induced neurodegeneration through mechanisms not completely defined. Here, we investigated the neuroprotective effect of Arc on H89-induced cell damage and its potential mechanisms in mouse cortical neurons and human SH-SY5Y neuroblastoma cells. We found that Arc prevented cell viability loss induced by H89 in human SH-SY5Y cells. Moreover, Arc reduced intracellular beta amyloid (Aβ) production induced by H89 in neurons and human SH-SY5Y cells, and Arc also inhibited the presenilin 1(PS1) protein level in neurons. In addition, neural apoptosis in both types of cells, inhibition of neurite outgrowth in human SH-SY5Y cells and reduction of synaptic marker synaptophysin (SYN) expression in neurons were also observed after H89 exposure. All these effects induced by H89 were markedly reversed by Arc treatment. Arc also significantly attenuated downregulation of the phosphorylation of CREB (p-CREB) induced by H89, which may contribute to the neuroprotective effects of Arc. These results demonstrated that Arc exerted the ability to protect neurons and SH-SY5Y cells against H89-induced cell injury via upregulation of p-CREB.

## 1. Introduction

Arctigenin (Arc) is the main active constituent that is extracted and isolated from the fruit of *Arctium lappa* L., which has been used as an herbal medicine for its antipyretic and anti-inflammatory actions. Arc itself has antioxidant, anti-inflammatory, anti-tumor and immunomodulatory effects [1–3]. Moreover, Arc exerts a neuroprotective effect on both glutamate-induced neurotoxicity in primary neurons and scopolamine-induced learning and memory deficits in mice with Alzheimer’s disease (AD) [4,5]. However, the underlying mechanism remains elusive.

Beta-amyloid (Aβ) peptide is the central player in the amyloid cascade hypothesis that describes AD etiology [6]. Cyclic AMP response element-binding protein (CREB) is a nuclear transcription factor that is activated by phosphorylation at serine 133 [7]. Activation of CREB is essential for the formation and retention of memory, which is the main physiological parameter of Alzheimer’s disease [8]. The process of CREB activation is also considered to be a major mechanism in the promotion of neuronal growth and survival [9]. Reduced phosphorylation of CREB (p-CREB), which is considered to be one of the consequences of Aβ-induced neurotoxicity, has been observed in the postmortem brains of AD patients, Aβ-treated neurons [10] and in Tg-AD mice that overexpress Aβ [11]. CREB can be activated by multiple signaling pathways, including protein kinase A (PKA). H89, a selective pharmacological PKA inhibitor, possesses the ability to suppress CREB phosphorylation in a concentration-dependent manner [12] and, thus, has been widely used as a laboratory agent in inhibition of CREB phosphorylation [13–17]. Recent studies have reported that H89 reduced the viability of primary cortical neurons [18]. In the present study, we thus used H89 to induce neural cell damage via downregulation of p-CREB [19,20] and investigated if Arc protects neural cells against CREB inactivation.

## 2. Results and Discussion

### 2.1. Arc Protects Human SH-SY5Y Cells from H89-Induced Reduction of Cell Viability

**Figure 1:**
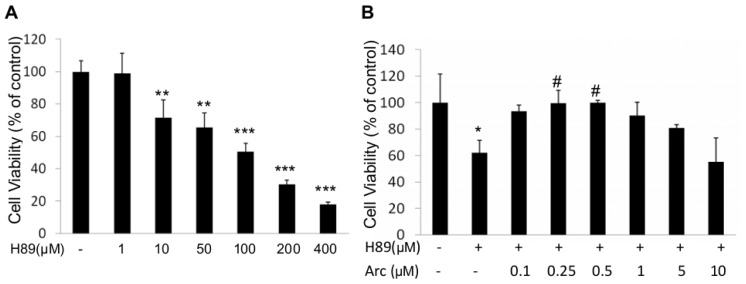
Protective effects of arctigenin (Arc) on human SH-SY5Y cells from H89-induced cell injury. (**A**) Human SH-SY5Y cells viability treated with different concentrations of H89 for 1 h; (**B**) Viability of cells treated with different concentrations of Arc after being exposed to H89 (50 μM) for 1 h. Cell viability was detected by MTT assay. Values represent the mean ± SD from three separate experiments (*n* = 9). *p < 0.05; **p < 0.01; ***p < 0.001 *vs.* the control; # *p* < 0.05 *vs.* the H89 group.

We first did a dose-response study of H89 on human SH-SY5Y cells. Cells were incubated with various concentrations of H89 (1–400 μM) for 1 h. Cell survival was assessed by MTT (3-(4,5-dimethyl-2-thiazolyl)-2,5-diphenyl-2-tetrazolium bromide) assay. H89 (10–400 μM) resulted in 28%–82% cell death (Figure 1A). Furthermore, H89 has been reported to induce downregulation of p-CREB at 50 μM [20]. We therefore used this dose in the next experiments.

To investigate whether Arc could rescue the loss of cell viability, human SH-SY5Y cells were treated with H89 (50 μM, 1 h) before being incubated with Arc (0.25–10 μM for 24 h). Cell viability was assessed by MTT assay. Results showed that the viability of H89-treated cells was reduced to 61.90% ± 9.79% in comparison with the control group (100%, *p* < 0.05, Figure 1B); Arc significantly increased the viability, with a maximal effect at 0.5 μM (99.86% ± 1.75%) (*vs*. H89-control, *p* < 0.05, Figure 1B). Arc decreased cell viability at concentrations higher than 1 μM, indicated that Arc exerted a neuroprotective effect at a proper range of concentrations (0.25–0.5 μM) against H89-induced cell damage.

### 2.2. Arc Attenuates Aβ Production Induced by H89

**Figure 2:**
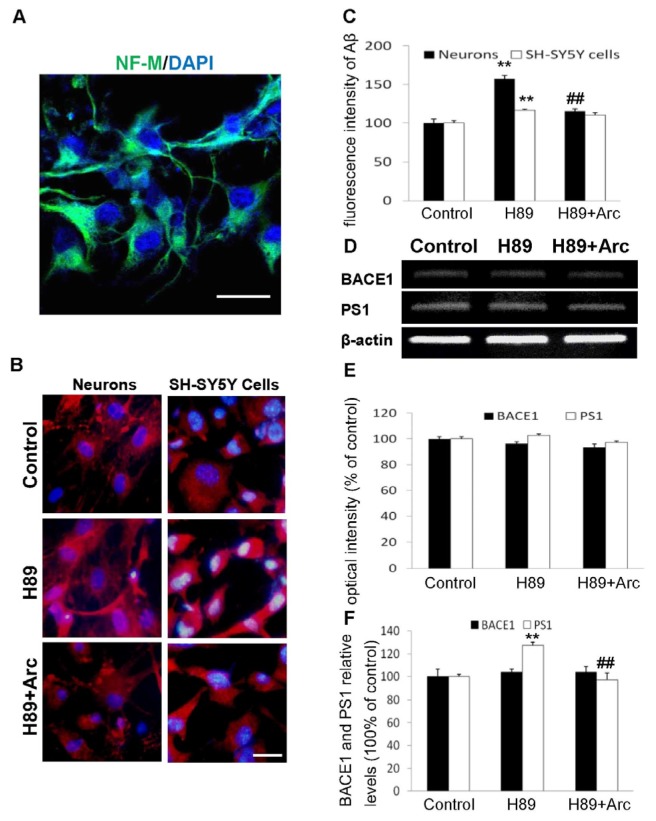
Arc attenuated Aβ35–42 production induced by H89 by reducing the PS1 protein level. (**A**) Neurons identified by immunostaining of neural marker, NF-M (green), and 4′,6-diamidino-2-phenylindole (DAPI, blue). Scale bar = 25 μm; (**B**) Neurons and SH-SY5Y cells were immunostained using anti-Aβ35–42 antibody (red) and DAPI (blue). Scale bar = 20 μm; (**C**) Average fluorescence intensity of Aβ immunostaining was measured in triple cultures; (**D**) mRNA of BACE1 and PS1 were analyzed by RT-PCR; (**E**) Quantitative analysis of the relative mRNA levels of BACE1 and PS1 by Image J software; (**F**) Cell supernatants of neurons were assayed using ELISA. The data were expressed as the mean ± SD (*n* = 6); **p < 0.01 *vs.* the control; ## *p* < 0.01 *vs.* the H89 group.

Mouse cortical neurons were cultured and identified with immunostaining of neural marker, Neurofilament M (NF-M) (Figure 2A). Aβ(35–42) expression in neurons and SH-SY5Y cells was determined by immunostaining with anti-Aβ(35–42) antibody. As shown in Figure 2B, the immunoreactivity of Aβ in H89-treated groups was greater than control groups, whereas it was significantly decreased after treatment with Arc for 24 h. Quantitative analysis of the immunocytochemistry suggested that the levels of Aβ in H89-treated neurons and SH-SY5Y cells were 156.93% ± 4.65% and 116.74% ± 1.61% of the control group, respectively (Figure 2C, *p* < 0.01, *p* < 0.01, *vs.* control 100%). Arc treatment attenuated the H89-induced increase of Aβ to 115.42% ± 3.29% and 110.70% ± 3.28%, respectively (Figure 2C, *p* < 0.01, *p* > 0.05, *vs.* H89 group).

We further investigated BACE1 and PS1 production by RT-PCR and ELISA. The results indicated that the mRNA expression of BACE1 and PS1 was not affected by H89 and H89 plus Arc treatment (Figure 2D,E) and with a similar protein level pattern of BACE1 (Figure 2F). However, the protein level of PS1 assayed by ELISA was significantly increased by H89 exposure, which was significantly reversed by the treatment with Arc (Figure 2F, 126.24% ± 2.94% of the H89 group *vs.* control, 100%, *p* < 0.01; 96.98% ± 6.44% of the H89 + Arc group *vs.* 126.24% ± 2.94% of the H89 group, *p* < 0.01). These data indicated that the effect of neurons incubated with Arc decreased the Aβ level induced by H89, which was not associated with significant changes in mRNA and the protein levels of BACE1. By contrast, the effect of Aβ inhibition was associated with a reduction of the PS1 protein level, indicating that the effect of Arc on the reduction of intracellular Aβ may be associated with the reduction of the PS1 protein.

### 2.3. Arc Protects Neuron-Like Cells and Neurons against Apoptosis Induced by H89

**Figure 3:**
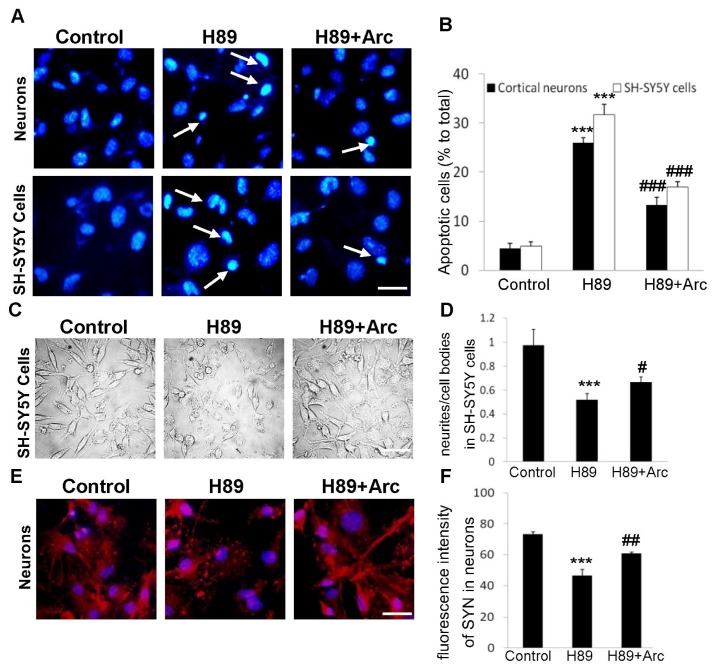
Arc inhibited apoptosis induced by H89 and restored neurite outgrowth and synaptic markers expression against H89-induced disorders. (**A**) Hoechst33258 staining was performed in mouse cortical neurons and SH-SY5Y cells. Scale bar = 25 μm; (**B**) Quantitative analysis of cell apoptosis; (**C**) Morphological characteristic of SH-SY5Y cells were observed using phase-contrast microscopy. Scale bar = 25 μm; (**D**) In 10 random fields, the number of neurites was counted, and the ratio of neurites to cell bodies was calculated; (**E**) Neurons were immunostained with synaptophysin (SYN, red) and DAPI (blue). Scale bar = 20 μm; (**F**) Average fluorescence intensity of SYN immunostaining was assessed in 10 random areas. All the data were expressed as the mean ± SD (*n* = 9); ***p < 0.001 *vs.* the control; # *p* < 0.05; ## *p* < 0.01; ### *p* < 0.001 *vs.* the H89 group.

To evaluate whether Arc protected neural cells form apoptosis, Hoechst33258 staining was performed, both in cortical neurons and SH-SY5Y cells (Figure 3A). The cells were incubated with H89 (50 μM) for 1 h prior to exposure to Arc (0.5 μM) for 24 h. The control groups showed intact and relatively large nuclei, whereas H89-treated cells showed an increase in condensed nuclei. Arc treatment reduced the number of condensed nuclei significantly compared with the H89 groups. Quantitative analysis showed that the apoptotic rate was 31.67% ± 2.08% in SH-SY5Y cells treated with H89 (*p* < 0.001, compared with the control, Figure 3B) and 17.07% ± 1.25%, when cells were cultured with Arc (*p* < 0.001, compared with the H89 group). Meanwhile, the percentage of apoptotic neurons was 26.79% ± 1.49% in neurons in the H89 group (*p* < 0.001, compared with the control) and 13.33% ± 1.53%, when neurons were incubated with Arc (*p* < 0.001, compared with the H89 group). All these results indicated that Arc effectively protected neuron-like cells and cortical neurons against H89-induced apoptosis.

### 2.4. Arc Restores Neurite Outgrowth and SYN Expression against H89-Induced Disorders

To assess the neuroprotection of Arc on synaptic impairment induced by H89, We first examined the neurite outgrowth in human SH-SY5Y cells. Cells were incubated with Arc (0.5 μM, 24 h) after being treated with H89 for 1 h. For quantification of neurite outgrowth, cell morphology was observed using phase-contrast microscopy. The number of neurites was counted, and the ratio of neurites to cell bodies was calculated. H89-treated cells displayed polygonal cell bodies and short processes. Cells incubated with Arc significantly reversed the appearance of neurite short processes induced by H89 (Figure 3C). As shown in Figure 3D, the ratio of neurites to cell bodies in H89-treated cells significantly decreased by 51.80% ± 5.48% of the control group (*p* < 0.001), while Arc-treated cells exhibited a higher value compared to the H89-treated cells (64.78% ± 1.31% *vs.* 51.80% ± 5.48%, *p* < 0.05).

In neurons, we performed an analysis of immunocytochemical staining of SYN, which is an integral membrane glycoprotein of synaptic vesicles, which has been widely used as a synaptic marker to investigate synaptic reinnervation and synaptogenesis [21]. The immunostaining of SYN was markedly decreased in the H89-treated neurons (Figure 3E,F, *p* < 0.001, compared with the control), indicating the occurrence of synaptic degeneration. However, the decrease of SYN induced by H89 was greatly alleviated after Arc treatment (Figure 3E,F, *p* < 0.01). Moreover, we treated neurons and SH-SY5Y cells with Arc in the absence of H89, and we found that there was no difference between the control groups and Arc (0.5 μM) alone (data not shown). These findings suggested that Arc protected neural cells against H89-induced synaptic impairment.

### 2.5. CREB May Be Involved in the Neuroprotection of Arc against H89-Induced Cell Injury

**Figure 4:**
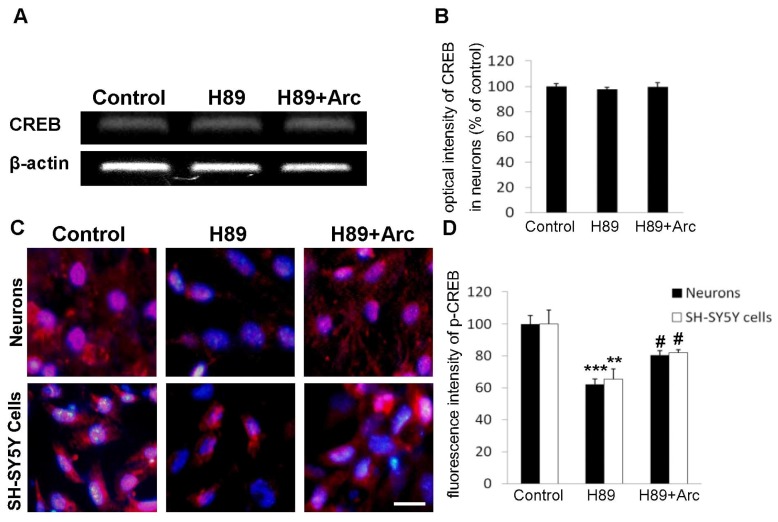
Arc attenuated the inhibition of the phosphorylation of cyclic AMP response element-binding protein (CREB) (p-CREB) induced by H89 in SH-SY5Y cells and neurons. (**A**) The mRNA of CREB was analyzed by RT-PCR in neurons; (**B**) The relative optical density of CREB mRNA was acquired by Image J (NIH Image J 1.38×, National Institutes of Health, Bethesda, MD, USA); (**C**) Neurons and SH-SY5Y cells were immunostained for p-CREB (red) and DAPI (blue). Scale bar = 20 μm; (**D**) The average fluorescence intensity of p-CREB immunostaining was assessed in 10 random areas. All the data were expressed as the mean ± SD (*n* = 3); **p < 0.01; ***p < 0.001 *vs.* the control; # *p <* 0.05 *vs.* the H89 group.

We then studied the correlation between CREB phosphorylation and the neuroprotective potential of Arc in neural cells. The expression of CREB mRNA was first assessed following amplification of RNA isolated from neurons. There was no significant change in the mRNA level of CREB in neurons-treated with H89 compared with the control, while cells incubated with Arc showed an increase (not significant) in the level of CREB mRNA (Figure 4A,B). Next, immunocytochemistry was performed to visualize the activation of CREB by Arc in neurons and human SH-SY5Y cells. H89-treated cells expressed most of the p-CREB in their cytoplasm, and the addition of Arc (0.5 μM) resulted in the translocation of p-CREB immunoreactivity into the nuclear region (Figure 4C). As shown in Figure 4D, treatment with H89 led to a decrease of the density of immunoreactive p-CREB in both cortical neurons (62.30% ± 4.06%, compared with the control, *p* < 0.001) and SH-SY5Y cells (65.46% ± 6.46%, compared with the control, *p* < 0.01). Whereas the effects were significantly alleviated by treatment with Arc in neurons (80.57% ± 3.34%, compared with the H89 group, *p* < 0.05) and SH-SY5Y cells (82.04% ± 1.98%, compared with the H89 group, *p* < 0.05). These data suggested that Arc may upregulate CREB, which contributes to its neuroprotective activity.

### 2.6. Discussion

In this study, we demonstrated for the first time that Arc attenuation of H89-induced enhancement of Aβ production in primary neurons and SH-SY5Y cells reduced PS1 protein expression in neurons. Moreover, Arc curtailed H89-induced cell viability inhibition, neural apoptosis and synaptic impairment, which may be associated with the reduction of H89-induced CREB inactivation, and exerted neuroprotective effects.

The presence of Aβ has been implicated as one of the most important pathogenic traits of AD. Previous studies have demonstrated that Aβ induces an inflammatory response, oxidative stress, mitochondrial dysfunction and neural apoptosis, resulting in neurodegeneration [22,23]. Therefore, reducing the level of Aβ in neurons could have profound effects on AD pathophysiology. Here, we found that exposure of SH-SY5Y cells and primary neurons to H89 elicited a significant increase in intracellular Aβ. The H89-induced increase in Aβ was attenuated by treatment with Arc, showing that Arc can block this effect of H89 on Aβ levels.

Aβ is derived from the cleavage of amyloid precursor protein (APP). APP undergoes ectodomain shedding at two alternative sites; one is mediated by BACE1 and results in the formation of membrane-associated *C*-terminal fragments (CTFs), termed C99s. The subsequent PS/γ-secretase-mediated cleavage of C99 at the γ-site releases Aβ peptides of different lengths [24]. The ability of Arc to depress the H89-induced increase in Aβ suggests that Arc may play a role in Aβ production during APP processing. Consistent with this hypothesis, we observed that while the mRNA levels of BACE1 and PS1 were not altered in mouse primary neurons exposed to H89 or H89 plus Arc, the PS1 protein level was significantly increased by H89 and reversed by Arc treatment, suggesting that Arc reduced Aβ production by decreasing the PS1 protein level. It should be noted that the reduction in Aβ levels by Arc was only associated with the PS1 protein, and not mRNA, levels. Thus, how Arc reduces PS1 protein expression warrants further investigation.

In the present study, we also found that Arc treatment could inhibit H89-induced depression of cell viability and neural apoptosis in both types of cells (cortical neurons and SH-SY5Y cells), thereby rescuing them from neurodegeneration and confirming the neuroprotective action of Arc against H89. It is known that synaptic impairment and synapse loss are events in the early stages of AD [25]. Therefore, restoring synaptic integration in neurons could be beneficial for the treatment of AD. Previous studies have shown that H89 robustly inhibits neurite formation in PC12 cells [26], and another PKA inhibitor is shown to dramatically suppress the levels of SYN in neurons [27]. Here, we found that neurite outgrowth was significantly inhibited by H89 in SH-SY5Y cells and that the expression of SYN was decreased by H89 in cortical neurons. Moreover, administration of Arc to the two types of cells studied resulted in a significant alleviation of the H89-induced reduction in neurite outgrowth in SH-SY5Y cells and of the H89-induced disturbance of SYN expression, further demonstrating the neuroprotective effect of Arc.

In addition to its roles in other physiological processes, including Long-term Potentiation (LTP), CREB can be activated by PKA [28] to regulate the transcription of an array of pro-survival and anti-apoptotic genes [29]. Thus, it is not surprising that the consequences of disrupting CREB function are severe. H89 is a selective PKA inhibitor that has been reported to significantly inhibit the activation of CREB [12]. In this study, we first examined the mRNA level of CREB and found that H89 and Arc did not alter the mRNA expression. We further performed immunocytochemistry to visualize p-CREB; we found that exposure to H89 elicited a significant inhibition of CREB phosphorylation in both cortical neurons and SH-SY5Y cells. Administration of Arc reversed the H89-induced downregulation of p-CREB in these cells. However, further experiments, such as Western blot, should perform to attain the ratio of pCREB/CREB to confirm the results. Collectively, these results indicate that restoration of CREB phosphorylation by Arc in H89-treated cells accounts for the neuroprotective effects of Arc.

## 3. Experimental Section

### 3.1. Cell Culture

Primary cortical neurons were prepared from neonatal (P0–P2) mouse brain using methods described previously [30]. A neuron-like cell line, human SH-SY5Y cells, was plated at a density of 1.0 × 10^5^/mL. The cultures were grown in Dulbecco’s modified Eagle’s medium (DMEM) supplemented with 10% fetal bovine serum (FBS), 100 U/mL penicillin and 100 μg/mL streptomycin (all from Gibco, New York, NY, USA) at 37 °C in a humidified atmosphere of 5% CO_2_.

### 3.2. Preparation of Arc

Arc was purchased from the National Institute for the Control of Pharmaceutical and Biological Products (Beijing, China; molecular formula: C_21_H_24_O_6_; molecular weight: 372.4117) and kept from direct exposure to light and air during the experiments. Stock solution of Arc (1 mM) was dissolved in Phosphate Buffered Saline (PBS) and stored at −20 °C.

### 3.3. Cell Viability

Human SH-SY5Y cells were pretreated with H89 for 1 h, followed by exposure to Arc (0.5 μM) for 24 h. Cell viability was measured by MTT assay. Briefly, cells were incubated with 0.5 mg/mL MTT (Sigma, St. Louis, MO, USA) at room temperature (RT) for 4 h. Formed formazan crystals were dissolved in Dimethyl sulfoxide (DMSO), and the plates were analyzed using a microplate reader (MR-96A, Mindray, Shenzhen, China) at 540 nm.

### 3.4. Immunofluorescence, Hoechst33258 Staining and Neuritogenesis

Cells were fixed for 30 min with 4% paraformaldehyde and washed three times with PBS. The cells were incubated with primary antibody at 4 °C overnight. The primary antibodies used were as follows: mouse anti-NF-M (1:150; StemCell Technologies, Vancouver, BC, Canada), rabbit anti-SYN1 (1:100), rabbit anti-beta-amyloid (35–42) (1:100) and rabbit anti-p-CREB (1:100; all from Bioss. Inc.; Beijing, China). After rinsing with PBS, cells were incubated with appropriate Cy3, fluorescein isothiocyanate (FITC)-conjugated species-specific secondary antibodies (1:200; all from Jackson ImmunoResearch Lab, West Grove, PA, USA) at RT for 1 h. The cells were mounted with mounting medium (Vector Laboratories, Burlingame, CA, USA) containing DAPI or Hoechst 33258 (0.5 μg/mL; Sigma, St. Louis, MO, USA). Fluorescence was recorded on an inverted fluorescence microscope (NOVEL NIB-100F, NOVEL, Beijing, China). Fluorescence intensities of SYN, Aβ and p-CREB were measured using Image J software (NIH Image J 1.38×, National Institutes of Health, Bethesda, MD, USA) [31]. Cell apoptosis was assessed by nuclear morphology. Cells with condensed chromatin of fragmented nuclei were counted as apoptotic cells. Cells were counted in triplicate cultures in ten randomly chosen areas under the microscope in 2–4 experiments. The cell counter of the Image J software (NIH Image J 1.38×, National Institutes of Health, Bethesda, MD, USA) was used to count cells, and mean numbers were used for analysis. The frequencies of apoptotic cells were expressed as the relative percentage per 100 cells [32]. For analysis of neuritogenesis, images were captured over randomly selected fields by using a 40× objective and constant camera settings within each experiment. At least 10 fields were analyzed for each sample. In each selected field, the numbers of cell bodies and neurites were counted [33].

### 3.5. Quantitative PCR with Reverse Transcription

For mRNA quantification, the total RNA of neurons was isolated using TRIzol reagent (Carlsbad, CA, USA). A RevertAid First Strand cDNA Synthesis Kit (Fermentas, Lafayette, CO, USA) was used to synthesize the cDNA. PCR was performed using the DreamTaq Green PCR Master Mix Kit (all from Thermo Scientific, Lafayette, CO, USA). The primers used to detect the expression of *CREB* were: 5′-ATA AAG CCT GCA ACA GCC AAC T-3′ (forward) and 5′-CAA AGA CCT GCT AAT CCT CAC G-3′ (reverse); *BACE*1 were: 5′-TAG GAT CCA TGG CCC CAG CGC TGC ACT-3′ (forward) and 5′-CGG AAT TCT TAC TTG ATC TAA-3′(reverse); *PS*1 were: 5′-CAA CCC TGA GCC AAT TCA CAA GA-3′ (forward) and 5′-CGG GTA TAG AAG CTG ACT GAT-3′(reverse); *β-actin* were: 5′-TGC TGT CCC TGT ATG CCT CT-3′ (forward) and 5′-TTT GAT GTC ACG CAC GAT TT-3′ (reverse). The PCR cycling program parameters were as follows: 30 s, 94 °C; 35 cycles of 30 s, 56 °C; 1 min, 72 °C. The final extension was separated by electrophoresis on 1.0% agarose gels. The values obtained for the target gene expression were normalized to β-actin and quantified relative to the expression in control samples.

### 3.6. Quantification of BACE1 and PS1 by ELISA

β-secretase (BACE1) and presenilin 1 (PS1) levels were quantified in cell supernatants by an ELISA kit (R & D) following the manufacturer’s instruction.

### 3.7. Statistical Analysis

Results were expressed as the mean ± SD from an appropriate number of experiments. Statistical evaluation was performed by one-way ANOVA with Bonferroni’s multiple comparison test.

## 4. Conclusions

This collective evidence indicates that compared to H89-treated mouse cortical neurons and human SH-SY5Y cells, cells treated with Arc plus H89 showed enhanced cell viability, decreased Aβ production, reduced PS1 protein levels, attenuated cell apoptosis, increased neurite outgrowth and expression of the synaptic marker, SYN, and the underlying mechanism may be attributed to the enhancement of CREB activation.
